# Increased stressor‐evoked cardiovascular reactivity is associated with reduced amygdala and hippocampus volume

**DOI:** 10.1111/psyp.13277

**Published:** 2018-08-22

**Authors:** Gavin P. Trotman, Peter J. Gianaros, Jet J. C. S. Veldhuijzen van Zanten, Sarah E. Williams, Annie T. Ginty

**Affiliations:** ^1^ School of Sport, Exercise and Rehabilitation Sciences University of Birmingham Birmingham United Kingdom; ^2^ Department of Psychology University of Pittsburgh Pittsburgh Pennsylvania; ^3^ Department of Psychology and Neuroscience Baylor University Waco Texas

**Keywords:** amygdala, brain morphology, hippocampus, individual differences, magnetic resonance imaging, psychological stress, stressor‐evoked cardiovascular reactivity

## Abstract

Exaggerated cardiovascular reactivity to acute psychological stress is associated with an increased risk of developing cardiovascular disease. The amygdala and hippocampus have been implicated in centrally mediating stressor‐evoked cardiovascular reactivity. However, little is known about the associations of amygdala and hippocampus morphology with stressor‐evoked cardiovascular reactivity. Forty (*M*_age_ = 19.05, *SD* = 0.22 years) healthy young women completed two separate testing sessions. Session 1 assessed multiple parameters of cardiovascular physiology at rest and during a validated psychological stress task (Paced Auditory Serial Addition Test), using electrocardiography, Doppler echocardiography, and blood pressure monitoring. In Session 2, 1 year later, structural MRI was conducted. Brain structural volumes were computed using automated segmentation methods. Regression analyses, following Benjamini‐Hochberg correction, showed that greater heart rate and cardiac output reactivity were associated with reduced amygdala and hippocampus gray matter volume. Systolic blood pressure reactivity was associated with reduced hippocampus volume. In contrast, no associations between diastolic blood pressure, mean arterial blood pressure, stroke volume, or total peripheral resistance reactivity with amygdala or hippocampus volumes were apparent. Comparison analyses examining insula volume found no significant associations. Some indicators of greater stressor‐evoked cardiovascular reactivity associate with reduced amygdala and hippocampus gray matter volume, but the mechanisms of this association warrant further study.

## 1 INTRODUCTION

The reactivity hypothesis postulates that exaggerated cardiovascular reactivity to acute psychological stress predisposes individuals to risk for cardiovascular disease (Manuck, Kasprowicz, & Muldoon, 1990; Obrist, 1981). Evidence consistent with this hypothesis has been derived from cross‐sectional and prospective studies showing that higher levels of stressor‐evoked cardiovascular reactivity are associated with future blood pressure status and hypertension (Carroll, Ginty, Painter, et al., 2012), atherosclerosis (Barnett, Spence, Manuck, & Jennings, 1997), and cardiovascular disease mortality (Carroll, Ginty, Der, et al., 2012). Indeed, a meta‐analysis of 36 studies showed greater stressor‐evoked cardiovascular reactivity to associate with adverse future cardiovascular health status (Chida & Steptoe, 2010).

Considerable effort has been put into examining the bases of stable individual differences in stressor‐evoked cardiovascular reactivity. Evidence from neuroimaging studies has extended prior animal evidence to implicate neural systems as partial mediators of individual differences in stressor‐evoked cardiovascular reactivity (Gianaros & Sheu, 2009; Gianaros & Wager, 2015; Gianaros et al., [Link]; Ginty, Kraynak, Fisher, & Gianaros, 2017; Wager, Ast, et al., 2009). For example, human neuroimaging studies demonstrate that the hippocampus and amygdala are limbic structures related to autonomic cardiovascular control during stress (e.g., Gianaros & Wager, 2015; McEwen & Gianaros, 2011), and a recent review has suggested that these brain structures may influence stressor‐evoked cardiovascular reactivity via predictive neural processes that calibrate physiology with anticipated behavioral demands (Gianaros & Jennings, 2018). Although it is known that stressor‐evoked cardiovascular reactivity is associated with concurrent activation in these corticolimbic systems (Gianaros & Sheu, 2009; McEwen & Gianaros, 2011; Myers, 2017; Wager, Waugh, et al., 2009), there is substantially less research examining if underlying morphological differences of the hippocampus and amygdala are associated with individual differences in cardiovascular stress reactivity. Recent work supports the importance of examining the relationship between brain morphology and cardiovascular autonomic function, where results have found high frequency heart rate variability (indicative of cardiac vagal function) to be negatively associated with gray matter volumes of striatal and limbic structures (e.g., amygdala) of the central autonomic network (Wei, Hong, & Wu, 2018).

To our knowledge, only one study has directly examined the associations between stressor‐evoked cardiovascular reactivity and brain morphology in humans (Gianaros et al., 2008). Thirty‐two healthy participants underwent a structural MRI scan. They then completed a standard stress task while functional activation of the brain and mean arterial pressure were measured simultaneously. Greater stressor‐evoked mean arterial pressure reactivity was associated with lower amygdala gray matter volume as well as greater amygdala activity. The relationship between amygdala activation and stressor‐evoked mean arterial pressure reactivity statistically depended on gray matter volume of the amygdala. Secondary analyses found greater stressor‐evoked mean arterial pressure reactivity also associated with areas extending into the hippocampus (Gianaros et al., 2008). Thus, the amygdala and hippocampus volume appear to be involved in blood pressure reactivity to psychological stress. However, it is unknown if these associations are specific to blood pressure reactivity, or whether the hippocampus and amygdala are associated with other cardiovascular parameters.

The aim of the present study was to extend the previous study demonstrating greater stressor‐evoked pressure reactivity being associated with reduced amygdala and hippocampus gray matter volume (Gianaros et al., 2008) by including more comprehensive measures of cardiovascular activity (i.e., heart rate, stroke volume, cardiac output, total peripheral resistance, systolic and diastolic blood pressure, and mean arterial pressure). Additional analyses using a comparison region (the insula) were conducted to determine whether any significant associations were relatively specific to the amygdala and hippocampus. The insula was selected as the comparison region because it is functionally implicated in visceral control, specifically in the regulation of cardiovascular and autonomic function (Beissner, Meissner, Bar, & Napadow, 2013; Oppenheimer & Cechetto, 2016). Prior human imaging work, however, has not identified an association between insular morphology and stressor‐evoked cardiovascular reactivity. Therefore, it would not be expected to correlate with stressor‐evoked cardiovascular reactivity. We hypothesized that higher levels of stressor‐evoked cardiovascular reactivity would be associated with reduced volumes of the amygdala and hippocampus, but that there would no association between cardiovascular reactivity and insula volume.

## 2 METHODS

### 2.1 Participants

Participants were 40 healthy women (mean age = 19.05, *SD* = 0.22 years; see Table 1 for demographics), participating in the Stress and Transitions to University (STUN) study (Ginty, Brindle, & Carroll, 2015). The original STUN study included 81% women. The main aim of the STUN study was to examine how physiological responses to acute psychological stress predicted adaptation to college. A subsample of participants was recruited for this neuroimaging protocol. Only women were recruited for this small study using a subsample of the original sample due to the skew in gender in the original study. None of the participants had a history of cardiovascular disease or were smokers. All were asked to abstain from alcohol and vigorous exercise for 12 hr, caffeine for 2 hr, and food and drinks other than water for 1 hr before testing. All participants provided written consent prior to testing and received £10 for study participation. The study was approved by the University Ethics Committee.

**Table 1:** Mean (*SD*) participant demographics

	Mean (*SD*)
Age	19.05 (0.22)
BMI	23.13 (4.73)
	**No. (%)**
Ethnicity	
White	24 (60)
Black	4 (10)
Other	12 (30)
Socioeconomic status	
Professional	16 (40)
Managerial	8 (20)
Skilled nonmanual	7 (17.5)
Skilled manual	1 (2.5)
Partly skilled	3 (7.5)
Unskilled	5 (12.5)

### 2.2 Cardiovascular measurements

Heart rate (HR) was measured continuously by electrocardiography (ECG) with electrodes (CONMED Corp., Utica, NY) placed in a three‐lead configuration. Raw ECG data were collected using a Grass P511 amplifier (Grass Instruments, USA), CED Power1401 digital to analog converter, and Spike 2 software at a sampling frequency of 1,000 Hz. Using Kubios HRV, individual traces were visually inspected and any artifacts were removed. Average HR was calculated for each period (baseline, stress) using the full 10 min of data from each phase. Systolic blood pressure (SBP), diastolic blood pressure (DBP), and mean arterial pressure (MAP) were measured using a semiautomatic oscillatory blood pressure monitor (Dinamap Pro 100; Critikon Inc., Tampa, FL) at minutes 2, 4, 6, and 8 of baseline and task periods. Stroke volume (SV, ml) was assessed using a Phillips Sonos 7,500 ultrasound machine with an S3 two‐dimensional transducer (1–2 MHz). Flow through the aortic valve during systole was identified with an apical five‐chamber view of the heart using Doppler. The velocity profile of the aortic flow was obtained using pulsed‐wave spectral mode at a screen sweep speed of 100 mms^−1^. Doppler sampling of the flow was taken immediately below the orifice of the aortic valve. The flow was quantified automatically using the velocity time integral, which is the mean distance through which blood travels in the outflow tract during ventricular contraction. Each measurement of velocity time integral was made from at least three velocity profiles. The diameter of the aortic valve was measured from a parasternal long axis view, and the aortic valve area was calculated. Cardiac output (CO) was calculated as HR × SV and is reported in liters/min. Total peripheral resistance (TPR; dyne‐s/cm^‐5^) was calculated using the formula TPR = (MAP/CO) × 80.

### 2.3 Acute psychological stress task

Participants completed a 10‐min version of the Paced Auditory Serial Addition Test (PASAT; Gronwall, 1977) whereby a series of single‐digit numbers were presented through audio speakers. Participants were asked to add consecutive numbers together and verbalize their answers, while remembering the most recent number in order to add it to the next presented number. The PASAT involves social evaluation; participants were videotaped, and they were informed that “body language experts” would assess their anxiety levels. In reality, no such body language analysis took place. Additionally, a mirror was placed directly in front of the participant, and they were instructed to watch themselves for the duration of the test. Finally, participants were informed that they would hear a loud buzzer if they hesitated or answered incorrectly. Together, these elements of social evaluation, increased time pressure, and punishment have been shown to increase self‐reports of stress (Veldhuijzen Van Zanten et al., 2004). The PASAT reliably evokes cardiovascular reactivity (Ring, Burns, & Carroll, 2002; Veldhuijzen Van Zanten et al., 2004) and yields acceptable test‐retest reliability in multiple parameters of physiology (Ginty, Gianaros, Derbyshire, Phillips, & Carroll, 2013).

### 2.4 Procedures

One hundred and eighty‐five participants were briefed on the general protocol and provided informed consent after any questions had been answered. Firstly, a brief questionnaire pack was completed to collect demographic and other psychosocial information. Participants were asked to provide information about the occupational status of the parent who was the primary household provider, with options ranging from professional (e.g., doctor/lawyer) to unskilled worker (e.g., day laborer). Answers were then weighted from lowest to highest on a scale of 1 = *unskilled *to 6 = *professional*. This variable was taken to reflect parental socioeconomic status. Height and weight were assessed, and body mass index (BMI) was computed as weight (kg)/height (m)^2^. As part of the psychophysiology protocol, participants were asked to lie in a semilateral decubitus position on a hospital bed, with supportive pillows for comfort. ECG spot electrodes were attached, as well as a blood pressure cuff over the brachial artery. The Doppler echocardiography probe was positioned to check image quality. Following a 10‐min resting adaptation period, participants began a 10‐min resting baseline period where they remained quiet. Subsequently, participants were provided with instructions regarding the PASAT and completed a brief practice to ensure that they understood the task. Following this, all participants completed the 10‐min stress task, as well as a 10‐min recovery period (data not reported here). Finally, participants completed several questions regarding the perceived stressfulness and difficulty of the task, as well as their engagement. All questions were scored on a 7‐point Likert scale from 0 (*not at all*) to 6 (*extremely*). During Visit 2, approximately 1 year later, 40 participants were randomly selected to attend the Birmingham University Imaging Centre for a 1.5‐hr visit, during which structural MRI data were collected.

### 2.5 Acquisition of brain imaging data

Neuroimaging data were obtained using a Philips 3.0T Achieva system with 8‐channel head coil. Structural images were acquired using the T1 TFE (turbo field echo) technique (TR = 8.4 ms, TE = 3.8 ms, FoV = 232 mm, flip angle = 8° 288 × 288 matrix, 175 slices, voxel size 1 × 1 × 2 mm).

### 2.6 Image processing

The Freesurfer 5.3.0 software package (https://surfer.nmr.mgh.harvard.edu) was used to compute volumetric data (Fischl & Dale, 2000). Automated segmentation of the amygdala, hippocampus, and insula using this method has been previously described (Morey et al., 2009). Image preprocessing steps included affine registration to Talairach space, skull stripping, intensity bias correction, and spatial normalization. Data were inspected for outlier volume values. Outlier criteria were defined as any data that exceeded three standard deviations. Additionally, all data were visually inspected. Following visual and statistical inspection, no individuals exceeded our criteria. Subcortical structures were estimated by an automated segmentation and labeling method, based on a combination of structure location, voxel intensity, and spatial relationships with proximal subcortical structures (Fischl et al., 2002). Separate left and right hemisphere volumes were summed to provide total volumes for each area of interest. For each subject, an estimate of total intracranial volume (ICV) was obtained by a scaling factor from the Talairach transformation step (Buckner et al., 2004).

### 2.7 Data reduction and statistical analysis

Cardiovascular data were averaged for each 10‐min period (baseline, stress), and reactivity for each cardiovascular measurement was computed as the difference between baseline average and stress task average. Three participants did not have data for SV, CO, and TPR due to poor quality images being obtained with Doppler ultrasound. A repeated measures multivariate analysis of variance (MANOVA), using baseline and task cardiovascular values, was performed to confirm the stress task perturbed cardiovascular activity. Pillai's trace was reported as this is considered the most robust of the multivariate significance tests (Olson, 1976), and alpha level was set at *p < *0.05. To determine associations between stressor‐evoked cardiovascular reactivity and volumetric gray matter volume of amygdala, hippocampus, and insula regions, a series of two‐step hierarchical regressions was run. In all analyses, volumetric data were entered as dependent variables, and ICV, age, socioeconomic status (SES), and BMI were entered in Step 1 as covariates. Covariates were selected a priori based on previous literature (Gianaros et al., 2017) and as recommended to reduce bias in multiple regression models (Steyerberg, Eijkemans, Harrell, & Habbema, 2001). Cardiovascular variables were entered in Step 2, and separate analyses were run for each variable of interest owing to multicollinearity (HR, SBP, DBP, MAP, SV, CO, and TPR). Due to the large number of individual regressions, the Benjamini‐Hochberg (B‐H) method was implemented to reduce the false discovery rate and prevent the likelihood of a Type I error occurring (Benjamini & Hochberg, 1995). For each brain region, the *p* values of each regression are ranked and compared to computed B‐H critical values, which are calculated based on the number of tests run with a false discovery rate set at 0.05 (Benjamini & Hochberg, 1995). Significant results occur where *p* values from the regression are less than B‐H critical values. This method conservatively reduces the false discovery rate while power is maintained, thus effectively correcting for multiple comparisons (Benjamini & Hochberg, 1995; Thissen, Steinberg, & Kuang, 2002).

## 3 RESULTS

### 3.1 Stressor‐evoked cardiovascular reactivity and stress ratings

Participants rated the PASAT as moderately difficult (4.23 ± 0.92), stressful (4.68 ± 0.99), and engaging (4.13 ± 1.22), implying that the task was successful in inducing acute stress. A repeated measures MANOVA (baseline, stress) including all cardiovascular parameters revealed a significant multivariate time effect, Pillai's trace = 0.869, *F*(7, 30) = 28.496, *p* < 0.001, *η_p_* ^2^ = 0.869, observed power = 100%. Results examined at the univariate level revealed a significant difference in HR, SBP, DBP, SV, CO, MAP, and TPR. Post hoc analyses indicated that the stress task significantly increased HR, SBP, DBP, MAP, SV, and CO and decreased TPR compared to baseline (see Table 2).

**Table 2:** Mean (*SD*) values of cardiovascular variables during baseline and stress

	Mean (*SD*)					
	**Baseline**		**Stress**	*F*	*p*	*η* ^2^
Heart rate (bpm)	74.75 (12.63)		91.31 (14.57)	157.47	<0.001	0.814
Systolic blood pressure (mmHg)	109.03 (9.59)		121.98 (11.65)	163.86	<0.001	0.820
Diastolic blood pressure (mmHg)	67.78 (6.45)		76.99 (8.61)	107.50	<0.001	0.749
Mean arterial pressure (mmHg)	81.69 (7.50)		93.06 (9.38)	173.50	<0.001	0.828
Stroke volume (ml)	71.87 (10.15)		76.23 (11.86)	22.97	<0.001	0.390
Cardiac output (L/min)	5.20 (0.89)		6.94 (1.42)	119.25	<0.001	0.768
Total peripheral resistance (dyne‐s/cm^−^^5^)	1,290.89 (189.15)	1,117.05 (198.73)		50.06	<0.001	0.582

Note

Results are reported for repeated measures MANOVA univariate tests.

### 3.2 Structural volumes

The means for our structural volumes were as follows: hippocampus 7,945.31 ± 798.10 mm^3^, amygdala 3,235.51 ± 419.97 mm^3^, and insula 11,276.82 ± 1,247.15 mm^3^. The following correlations between brain volumes were found: hippocampus and amygdala volume, *r*(38) = 0.787, *p* < 0.001, hippocampus and insula, *r*(38) = 0.629, *p* < 0.001, and amygdala and insula, *r*(38) = 0.428, *p* = 0.005.

### 3.3 Stressor‐evoked cardiovascular reactivity and amygdala gray matter volume

**Figure 1:**
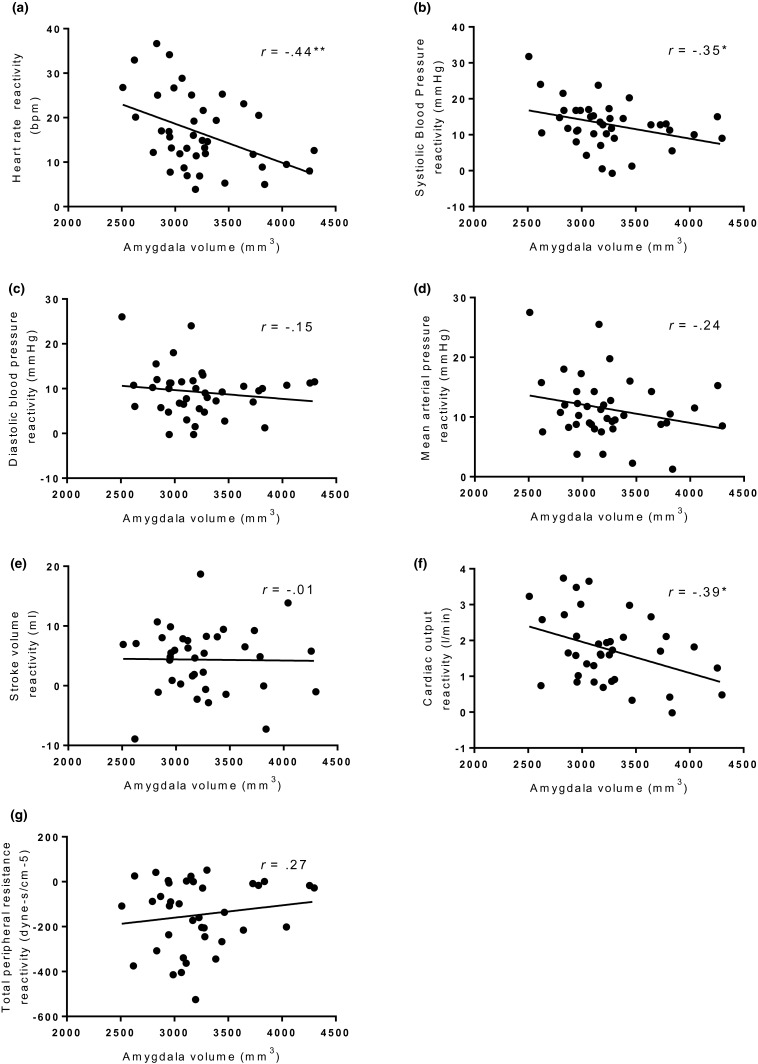
Scatterplots showing unadjusted amygdala volume plotted against (a) heart rate reactivity (b) systolic blood pressure reactivity (c) diastolic blood pressure reactivity (d) mean arterial pressure reactivity (e) stroke volume reactivity (f) cardiac output reactivity (g) total peripheral resistance reactivity. *p < 0.05, **p < 0.01

Seven separate regression analyses for each CVR variable were run. Stressor‐evoked HR reactivity (*β* = −0.476), CO reactivity (*β* = −0.445), and SBP reactivity (*β* = −0.357) significantly predicted total amygdala gray matter volume, when controlling for ICV age, SES, and BMI (see Table 3). Following B‐H correction, SBP reactivity was no longer significant. Thus, greater increases in HR and CO reactivity predicted lower gray matter volume of the amygdala. No significant associations following B‐H correction were evident for SBP, DBP, MAP, SV, or TPR reactivity with amygdala volume. See Figure 1 for unadjusted associations between amygdala gray matter volume and all cardiovascular reactivity variables.

**Table 3:** Regression coefficients and *p* values for regressions between stressor‐evoked cardiovascular reactivity and total amygdala, hippocampus, and insula volume controlling for intracranial volume, age, socioeconomic status, and body mass index

	Amygdala			Hippocampus			Insula		
	∆*R* ^2^	*β*	*p*	∆*R* ^2^	*β*	*p*	∆*R* ^2^	*β*	*p*
**∆**HR (bpm)	0.202	−0.476	0.002*	0.120	−0.367	0.008*	0.004	−0.067	0.597
**∆**SBP (mmHg)	0.106	−0.357	0.031	0.101	−0.350	0.016*	0.000	−0.006	0.964
**∆**DBP (mmHg)	0.016	−0.129	0.418	0.004	−0.067	0.638	0.012	0.111	0.363
**∆**MAP (mmHg)	0.056	−0.248	0.122	0.042	−0.214	0.132	0.000	0.020	0.874
**∆**SV (ml)	0.008	−0.095	0.584	0.019	−0.145	0.337	0.002	0.052	0.685
**∆**CO (L/min)	0.183	−0.445	0.006*	0.131	−0.376	0.008*	0.000	−0.018	0.889
**∆**TPR (dyne‐s/cm^−^^5^)	0.098	0.320	0.051	0.076	0.283	0.051	0.001	0.031	0.805

Note

Separate regressions were run for each cardiovascular variable. **∆**HR = heart rate reactivity; **∆**SBP = systolic blood pressure reactivity; **∆**DBP = diastolic blood pressure reactivity; **∆**SV = stroke volume reactivity; **∆**CO = cardiac output reactivity; **∆**MAP = mean arterial pressure reactivity; **∆**TPR = total peripheral resistance reactivity.

*Remains significant after adjusting for Benjamini‐Hochberg correction false discovery rate (FDR) = 0.05.

### 3.4 Stressor‐evoked cardiovascular reactivity and hippocampus gray matter volume

**Figure 2:**
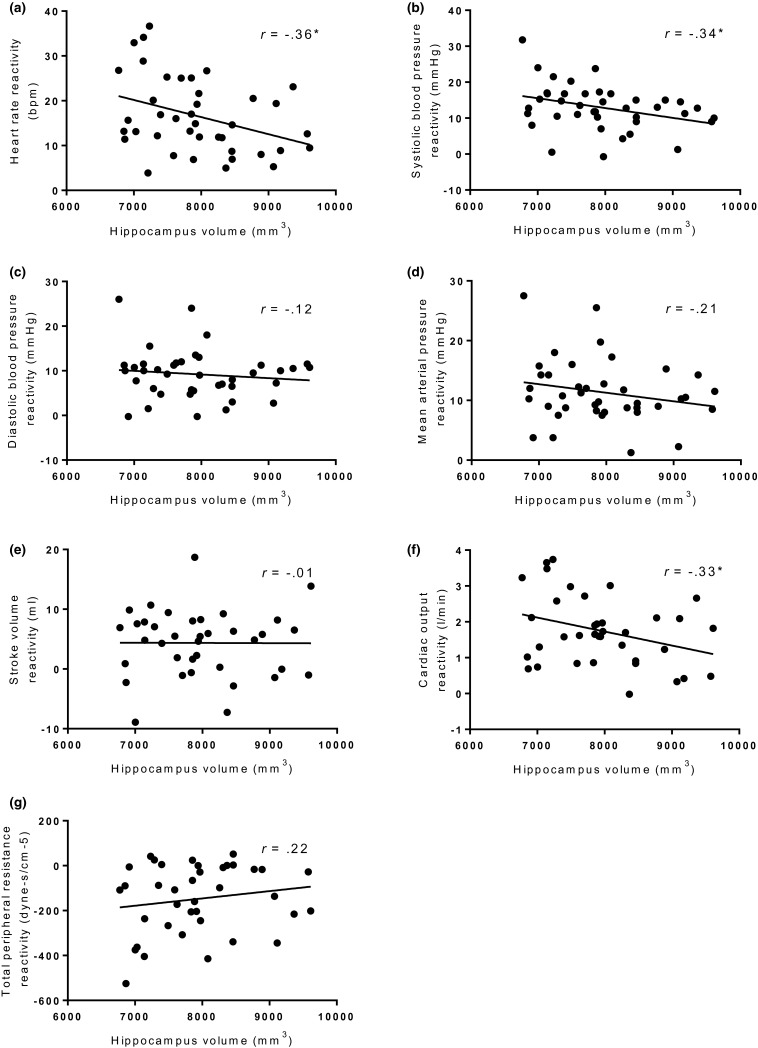
Scatterplots showing unadjusted hippocampus volume plotted against (a) heart rate reactivity (b) systolic blood pressure reactivity (c) diastolic blood pressure reactivity (d) mean arterial pressure reactivity (e) stroke volume reactivity (f) cardiac output reactivity (g) total peripheral resistance reactivity. *p < 0.05

Separate regression analyses found HR reactivity (*β* = −0.367), CO reactivity (*β* = −0.376), and SBP reactivity (*β* = −0.350) significantly predicted total hippocampus gray matter volume when controlling for ICV, age, SES, and BMI (see Table 3). Significant results remained following B‐H correction. Specifically, greater increases in HR, SBP, and CO reactivity predicted lower gray matter volume of the hippocampus. No significant associations were evident with DBP, MAP, SV, or TPR reactivity and hippocampus volume. See Figure 2 for unadjusted associations between hippocampus gray matter volume and all cardiovascular reactivity variables.

### 3.5 Stressor‐evoked cardiovascular reactivity and insula gray matter volume

**Figure 3:**
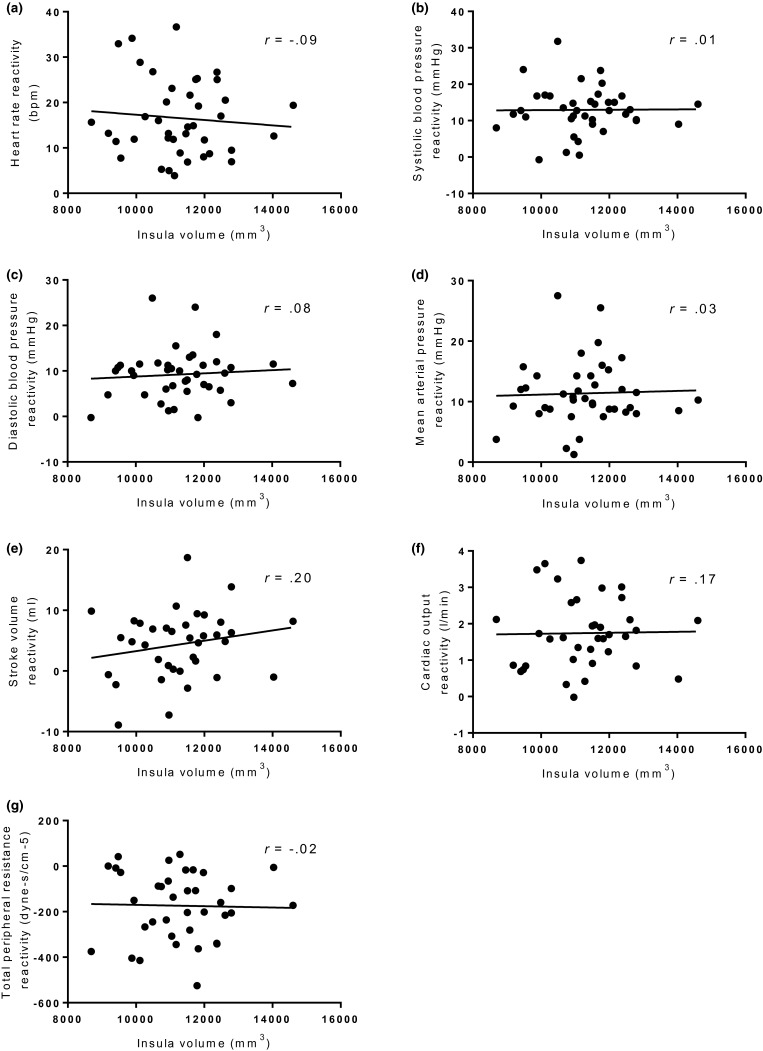
Scatterplots showing unadjusted insula volume plotted against (a) heart rate reactivity (b) systolic blood pressure reactivity (c) diastolic blood pressure reactivity (d) mean arterial pressure reactivity (e) stroke volume reactivity (f) cardiac output reactivity (g) total peripheral resistance reactivity

Seven separate regression analyses controlling for ICV, age, SES, and BMI found no significant relationships between CVR parameters and insula gray matter volume (see Figure 3 for unadjusted associations between insula gray matter volume and all cardiovascular reactivity values). In addition, B‐H correction did not alter the results (see Table 3).

## 4 DISCUSSION

This study examined the relationship between stressor‐evoked cardiovascular reactivity and amygdala and hippocampal volume. In line with our hypotheses, increased stressor‐evoked cardiovascular reactivity was associated with reduced amygdala and hippocampal volume but was not associated with insula gray matter volume. Specifically, greater HR, CO, and SBP reactivity were associated with reduced hippocampus, and HR and CO reactivity were associated with reduced amygdala volume. There were no associations between DBP, MAP, SV, nor TPR and amygdala or hippocampus gray matter volume. The finding that cardiovascular reactivity does not relate to insula volume, an area implicated in visceral control (Beissner et al., 2013; Oppenheimer & Cechetto, 2016), provides evidence that our observed relationship between cardiovascular reactivity and brain morphology may be more closely—although not exclusively—linked to medial temporal areas that are thought to influence peripheral stress physiology, namely, amygdala and hippocampus. Whole‐brain computational approaches that integrate other aspects of brain tissue morphology and cortical and subcortical areas are needed to fully explore this possibility.

In contrast with the only other human study explicitly examining the associations between stressor‐evoked cardiovascular reactivity with brain morphology, where MAP was associated with reduced amygdala and hippocampal gray matter volume (Gianaros et al., 2008), we found no associations between brain morphology and MAP reactivity. Several reasons could underlie the discrepancies between our MAP results and Gianaros et al. (2008). First, we measured cardiovascular parameters in a laboratory setting, whereas Gianaros and colleagues measured MAP while concurrently completing the fMRI protocol in the scanner. Consequently, participants were in a different postural position while completing the stress task, and these postural alterations could result in altered cardiovascular responses to the stressor (Sherwood & Turner, 1993; Turner, 1994). Second, although both studies employed validated stressors, the stress tasks were different. Different stress tasks are known to produce distinctive underlying hemodynamic responses that ultimately lead to blood pressure changes (Kasprowicz, Manuck, Malkoff, & Krantz, 1990). The present study sample experienced a greater increase in MAP compared to Gianaros et al. (2008), suggesting that the different tasks may account for the discrepancy in the results. Finally, while both studies computed amygdala and hippocampus volumes with validated automated segmentation methods (Dale, Fischl, & Sereno, 1999; Whitwell, 2009), the software used for automated segmentation differed across studies and could possibly underlie the discordance across the studies (Grimm et al., 2015). The current study used FreeSurfer, and the Gianaros et al. (2008) study used voxel‐based morphometry.

An interesting observation is that HR and CO reactivity (SBP reactivity prior to B‐H correction), but not DBP, TPR, SV, or MAP reactivity, relate to amygdala and hippocampus volumes. While the former group of cardiovascular variables are suggested to be cardiac driven and the latter group more vascular driven (Kasprowicz et al., 1990; Llabre, Klein, Saab, McCalla, & Schneiderman, 1998; Sherwood, Dolan, & Light, 1990), arguably it could be that more cardiac‐driven responses to psychological stress, rather than vascular responses, may more strongly be associated with reduced volumes of the amygdala and hippocampus. It is possible that, while Gianaros et al. (2008) found associations with MAP, this is a cardiovascular end point, and our study's more extensive cardiovascular parameters highlight that cardiac variables may be underlying this association.

The hippocampus and amygdala are rich with glucocorticoid receptors, which are low‐affinity receptors and are thus bound during periods of high glucocorticoid levels such as during acute stress (Jacobson & Sapolsky, 1991; Wang et al., 2014). Although the present study did not measure markers of the hypothalamus‐pituitary‐adrenal (HPA) axis, greater adrenocorticotrophic hormone and cortisol responses are associated with greater stressor‐evoked cardiovascular reactivity (Al'Absi, 1997), and cardiovascular reactivity to acute psychological stress has been shown to be greatly enhanced by HPA activity (Herd, 1986; Walker, Best, Shackleton, Padfield, & Edwards, 1996). It may be that individuals with atrophied volume of medial temporal lobe regions, such as the hippocampus and amygdala, exhibit an impaired negative feedback of the HPA axis, which results in inefficient restraint over cardiovascular or related responses to stress (Boyle et al., 2005; Furay, Bruestle, & Herman, 2008). Human studies supporting this hypothesis include individuals with Cushing's disease (Starkman, Gebarski, Berent, & Schteingart, 1992), where individuals produce excessive levels of glucocorticoids and show reduced medial temporal lobe volumes, particularly in the hippocampus. Following surgical correction for the hypercortisolemia, the volumetric reductions are largely remediated (Starkman et al., 1999). Owing to the cross‐sectional design of the present study and lack of HPA measurements, we are unable to draw inferences about causal associations, temporal ordering of associations, or the role of glucocorticoids in linking medial temporal lobe volumes with stressor‐evoked cardiovascular reactivity. Accordingly, these suggestions warrant further study.

Secondly, the reactivity hypothesis states that greater individual stressor‐evoked cardiovascular reactivity is associated with increases in future blood pressure status (Carroll, Ginty, Der, et al., 2012). Evidence has shown that higher blood pressure levels are associated with brain volume reductions in later life, particularly in the hippocampus (Beauchet et al., 2013; Jennings et al., 2012). Thus, it is plausible that greater stressor‐evoked cardiovascular reactivity could have neurodegenerative effects over the lifespan, specifically causing atrophy of the hippocampus. The suggested explanation underlying the effects of hypertension on brain volume is that hypertension causes atherosclerosis, resulting in blood vessel diameter changes, and consequently reduced perfusion to capillary beds in the brain (Beauchet et al., 2013; Dai et al., 2008). As a result of this hypoperfusion, regions including the frontal lobe and hippocampus are at increased risk for atrophy (Cohen, 2007). However, this perspective has been challenged by recent findings showing that neural alterations precede prospective increases in blood pressure, leaving unresolved the question of whether blood pressure is in fact causal to brain alterations (Jennings et al., 2017). Notwithstanding, our study population comprised a very young and healthy group of women at low cardiovascular disease (CVD) risk. Therefore, it seems unlikely that brain volume reductions are a consequence of hypertension or CVD burden. Rather, it could be suggested that brain volume reductions might predispose individuals toward stress sensitivity and greater reactivity (Gilbertson et al., 2002).

To elaborate, there is evidence to suggest that reduced volumes of the hippocampus and amygdala are heritable and may predispose individuals to increased stress‐related responsiveness (Gianaros et al., 2008; Meyer‐Lindenberg et al., 2006; Morey et al., 2009; Pezawas et al., 2005). For example, Gilbertson et al. (2002), implementing a monozygotic twin design, found stronger evidence for heritability influencing hippocampal volumes than stress exposure in twins where one of the twins had combat‐exposed post‐traumatic stress disorder. Animal work has shown that hippocampal size was dependent on genetic heritability (54%), but not different postnatal conditions. Importantly, monkeys with small hippocampal volumes experienced relatively exaggerated cortisol responses to social stress (Lyons, Yang, Sawyer‐Glover, Moseley, & Schatzberg, 2001). This indicates that reduced hippocampal volume increases the vulnerability of exhibiting exaggerated physiological stress responses. Complementing the above studies, in healthy adults, classified into high and low perceived stress groups based on the perceived stress questionnaire, groups differed in their hippocampal volumes. The high perceived stress group exhibited smaller hippocampal volumes compared to the low stress group, and over a 5‐year period, neither the high nor low stress groups displayed changes in perceived stress or hippocampal volume (Lindgren, Bergdahl, & Nyberg, 2016). It was therefore concluded that because no changes were found over the 5 years, smaller hippocampal volumes may be a vulnerability factor, contributing to experiencing exaggerated stress reactions (Lindgren et al., 2016). Together, the above studies indicate that smaller hippocampal volumes appear more likely to be a result of dispositional and possibly heritable factors, rather than a consequence of continued exposure to stress. Indeed, individuals with smaller hippocampal volumes may be at risk for experiencing chronic psychological and physiological distress (van Rooij et al., 2015). However, it should be acknowledged that the current study has a small size; therefore, our results should be replicated in a larger sample.

In agreement with evidence implicating reduced volumes of the hippocampus as a vulnerability to developing stress‐related hyper‐responsivity, evidence also implicates the amygdala as an important structure leading to exaggerated stress response. Animal work reveals that mice strains with small basolateral volumes of the amygdala express augmented fear and cortisol responses compared to those with larger amygdala volumes (Yang et al., 2008). Moreover, mice strains with small basolateral amygdala display an exaggerated cortisol response when subjected to a stress condition, which was not apparent in the medium or large amygdala mice strains (Yang et al., 2008). Further evidence demonstrates that individuals carrying genetic variants with increased risk of developing stress‐related psychopathology exhibit pronounced amygdala volumetric reductions (Meyer‐Lindenberg et al., 2006; Pezawas et al., 2005). Importantly, hyper‐responsive amygdala activity during fear processing was coupled with reduced amygdala volume, suggesting that reduced amygdala volume may directly couple with hyper‐responsive amygdala activity (Meyer‐Lindenberg et al., 2006; Pezawas et al., 2005). This could potentially explain the relationship relating structural volume of the amygdala to stressor‐evoked physiological responses (Gianaros et al., 2008; Meyer‐Lindenberg et al., 2006; Pezawas et al., 2005). Thus, it may be that reduced gray matter volumes of the amygdala and hippocampus could be partly heritable indicators of stress‐related phenotypes.

The current study is not without limitations. First, due to the cross‐sectional nature of the study, cause and effect and temporal ordering cannot be determined. Second, the current study consisted of a small sample of only women. However, our analysis, which included B‐H correction for multiple comparison (a conservative method), in addition to reporting null findings with the insula as a comparison brain volume, arguably offers more confidence in the results. Despite this, the replication of this study's findings in a larger sample including men is essential. Third, brain morphology is known to undergo changes over the course of a year (Cahn et al., 2002); therefore, it must be acknowledged that the reported results may be less reliable than if the stress testing and structural scan occurred over a shorter retest period. However, research has shown cardiovascular reactivity is relatively stable over a 1‐month period (Kamarck, Jennings, Stewart, & Eddy, 1993) as well as over an 18‐year period (Hassellund, Flaa, Sandvik, Kjeldsen, & Rostrup, 2010). Fourth, the test‐retest reliability of amygdala volume using automated segmentation in Freesurfer has been found to be low (intraclass correlation coefficient 0.6; Morey et al., 2010). In contrast, cardiovascular responses to acute psychological stress are relatively stable across repeated testing sessions and with different tasks (Ginty et al., 2013). To overcome reliability issues inherent with small data sets, replication of our novel study results should be replicated in a larger sample. Lastly, other potential mediators of gray matter volume reductions have been implicated such as inflammation (Marsland, Gianaros, Abrarnowitch, Manuck, & Hariri, 2008; O'Donovan et al., 2015). Future research should include measures of inflammatory markers to explore the role that inflammation may have on the relationship between stress and limbic morphology.

In conclusion, the current study demonstrates that reduced volumes of the amygdala and hippocampus are associated with greater stressor‐evoked cardiovascular reactivity. Future longitudinal studies are needed to delineate the causal bases of associations between brain structure and stressor‐evoked cardiovascular reactivity, which is a known correlate and predictor of future cardiovascular risk.
